# Exploring the in vivo wound healing effects of a recombinant hemolin from the caterpillar *Lonomia obliqua*

**DOI:** 10.1186/s40409-016-0093-4

**Published:** 2016-12-21

**Authors:** Ana Claudia Sato, Rosemary Viola Bosch, Sonia Elisabete Alves Will, Miryam Paola Alvarez-Flores, Mauricio Barbugiani Goldfeder, Kerly Fernanda Mesquita Pasqualoto, Bárbara Athayde Vaz Galvão da Silva, Sonia Aparecida de Andrade, Ana Marisa Chudzinski-Tavassi

**Affiliations:** Laboratory of Biochemistry and Biophysics, Butantan Institute, Av. Vital Brasil 1500, São Paulo, SP 05503-900 Brazil

**Keywords:** Hemolin, rLosac, Wound healing, Collagen

## Abstract

**Background:**

Hemolin proteins are cell adhesion molecules from lepidopterans involved in a wide range of cell interactions concerning their adhesion properties. However, hemolin’s roles in cell proliferation and wound healing are not fully elucidated. It has been recently reported that rLosac, a recombinant hemolin from the caterpillar *Lonomia obliqua*, presents antiapoptotic activity and is capable of improving in vitro wound healing. Therefore, this study aimed to explore rLosac’s in vivo effects using a skin wound healing model in rats.

**Methods:**

Circular full-thickness wounds in the rat dorsum skin were treated either with rLosac, or with saline (control), allowing healing by keeping the wounds occluded and moist. During the wound healing, the following tissue regeneration parameters were evaluated: wound closure and collagen content. Furthermore, tissue sections were subjected to histological and immunohistochemical analyses.

**Results:**

The rLosac treatment has demonstrated its capacity to improve wound healing, as reflected in findings of a larger number of activated fibroblasts, proliferation of epithelial cells, increase of collagen type 1, and decrease of inflammatory infiltrate.

**Conclusion:**

The findings have indicated the rLosac protein as a very promising molecule for the development of new wound-healing formulations.

## Background

Wound healing is a complex biological process that involves several physiological events, such as hemostasis, inflammation, proliferation, and remodeling [1]. Dermal fibroblasts constitute the main cellular component of connective tissues and play a critical role in the healing process, not only in the production and remodeling of extracellular matrix (ECM) proteins, but also in the migration of keratinocytes that facilitate wound closure [2]. During the remodeling stage, extracellular matrix components, such as the collagen fiber produced by dermal fibroblasts, undergo changes for the restoration of disrupted components [2].

A variety of biological molecules appears to be involved in triggering and regulating the processes of wound healing and tissue repair. The interaction between ECM and cells, as well as the modulation of cell responses, have played important roles in dynamically regulating wound healing and in establishing the normal tissue morphology and function [1–3].

Interestingly, processes in insects related to development – namely the immune system, cell migration and wound healing – are regulated by ecdysteroid hormones, which, in turn, up-regulate multifunctional molecules, such as hemolin proteins [2]. Hemolins are bacteria-induced proteins sharing homology with neural cell adhesion molecules [3]. Of note, they are also highly expressed in the caterpillars’ epidermis, but, due to their adhesion properties, they have been related to the immune system processes [4, 5].

According to Li et al. [6], hemolins can be found in several lepidopterans including *Antheraeapernyi, Hyalophoracecropia, Manducasexta, Bombyxmori, Hyphantria Hyphantria, Lymantriadispar*, and display the following four structurally conserved motifs [6–8]: (i) KRLS motif in domain 2 (D2), which is related to the protein kinase phosphorylation dependent on adenosine 3',5'-cyclic monophosphate (cyclic AMP or cAMP) and guanosine 3',5'-cyclic monophosphate (cyclic GMP or cGMP); (ii) NRTS motif in domain 3 (D3), which corresponds to the potential N-glycosylation region; (iii) SGK motif in D3, which is related to the region of protein kinase C phosphorylation; and, (iv) KDG/KNG motif also in D3, which corresponds to the moiety related to cell adhesion. A KDG/KNG motif in D1 was also found, but is apparently unrelated to cell adhesion [6, 7]. Intriguingly, in *M. sexta*, the region structurally conserved in D3 is absent.

*Lonomia obliqua* Stuart factor activator (Losac) is a protein from the caterpillar *Lonomia obliqua* that belongs to the hemolin family. The therapeutic effects of native and recombinant version of Losac, such as cytoprotection and cell adhesion, have been studied by our research group [9–11]. Lately, we have also applied *in silico* approaches (molecular modeling, computational chemistry and chemometric methods) in order to establish the structure-property-function relationships as to the structural motif KDG in the D3 domain of hemolin proteins.

**Fig. 1:**
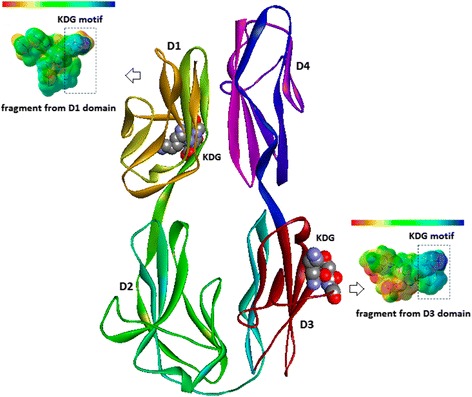
Losac three-dimensional molecular model shown as solid ribbons indicating the D3 (cell adhesion) and D1 domains. The KDG structural motifs are displayed as CPK or space-filling model (Discovery Studio Visualizer, v.4, Accelrys Software, Inc., 2005-2013). EP maps (B3LYP/3-21G*; Gaussian 03 W, Gaussian, Inc.; GaussView 0.5, Gaussian, Inc.) for the peptide fragments from D3 and D1 domains on the molecular surfaces were calculated, and can be interpreted by a color range from –0.11 (intense red; higher electronic density distribution) to +0.11 (intense blue; lower electronic density distribution)

The Losac three-dimensional (3D) molecular model, proposed by Alvarez-Flores et al. [10], is presented in Fig. 1. Domain 3, which displays the structural motif KDG related to cell adhesion, is presented in red and blue. Also, the map of electrostatic potential (EP) of a peptide fragment from that region is projected onto the molecular surface. Domain 1, which also has a structural motif KDG, but seems not to be related to cell adhesion, is highlighted in colors ranging from orange to green. The EP map of a peptide fragment from that region is also displayed on the molecular surface. Those maps can be interpreted in relation to a color range from intense red (negatively charged regions) to intense blue (positively charged regions). The differences in the electronic density distribution on the molecular surface of the peptide fragments are related to the structure-property-function relationships as to the amino acid substitution pattern in each domain (D1 and D3), especially those residues placed in the neighborhood of the KDG structural motif.

In relation to its biological activities, rLosac has induced cell proliferation and inhibited starvation-induced apoptosis in endothelial cells [9–11]. The rLosac is capable of protecting human fibroblast cells from apoptotic death induced by serum deprivation. Furthermore, during the stress condition induced by serum withdrawal, rLosac stimulates cells to produce extracellular matrix proteins, as well as to enhance the in vitro conditions related to wound healing [12]. Taking into account all the aforementioned information, we have evaluated herein the in vivo healing effects of rLosac using a skin full-thickness wound model in rats.

## Methods

### Recombinant protein

The recombinant protein (rLosac) was produced and purified, and its activity on factor X quantified as previously reported elsewhere [10].

### Full-thickness skin lesion model

Male Wistar rats aged 6 to 8 weeks and weighing 120 to 150 g were obtained at the Central Animal Breeding House, Butantan Institute. The animals were fed a standard pellet diet and water ad libitum. All procedures were approved by the Institutional Animal Care and Use Committee. Rats were anesthetized with a mixture of ketamine (75 mg/kg) and xylazine (10 mg/kg) administered intramuscularly. The dorsum was shaved and disinfected with ethanol. Four full-thickness excisional 4 sq mm punctures were cut through the skin (two wounds on each side), aseptically performed using a metallic puncher.

The lesions on the right side were topically treated with a single dose of rLosac (2.8 nM) whereas the wounds on the left side were treated with the vehicle (saline; control). Each wound was covered with a Bioclusive transparent bandage (Johnson & Johnson, USA). Full-thickness excisions were taken as a reference for normal intact skin, and considered the control. At intervals of 0, 3, 7, 14, and 21 days after wounding, the rats (n = 8, per group) were euthanized, and full-thickness skin samples from the healing wounds were excised for subsequent analyses. Before excision, the size of each lesion and the wounds’ contraction were evaluated using the KODAK In-Vivo Multispectral Imaging System FX and the Multispectral FX-Pro software. The data were presented as a percentage of the initial wound area.

### Image preparation and quantitative analysis

The software Multispectral FX-Pro allowed X-ray images to be co-registered, and to perform quantitative image analyses of the regions of interest. The analyses were performed on the control/treated lesions from each animal in the study protocol. The image intensity scale was kept constant, and the image intensity ratios were calculated for each time point image; mean ± SD was calculated and plotted as a function of image time.

### Histological analysis

Skin samples that comprised the section areas were removed and immediately incubated in formaldehyde (10%) buffer solution for 24 h. After that, the tissue was processed for histological analysis by a standard dehydration protocol, then degreased with xylene and embedded in paraffin. These samples were stored in paraffin blocks, and, then, 3-μm cuts were made using a histological microtome. They were kept in silanized glass slides, which were submitted to Hematoxylin-Eosin staining. The slides were examined under light microscopy in a Zeiss microscope coupled with an Image Evaluation System (Kontron 300).

### Histological and Immunohistochemical analyses

Immunohistochemistry for collagens type I 5-μm histological sections were deparaffinized, rehydrated and subjected to enzymatic digestion with 0.4% pepsin (Sigma, USA) diluted in 0.5 N acetic acid for 30 min at 37 °C. For proliferating cell nuclear antigen (PCNA) and α-smooth muscle actin (α-SMA), the 5-μm histological sections were deparaffinized, rehydrated and subjected to antigen retrieval in 10 mM sodium citrate buffer (pH 6.0) for 5 min in a pressure cooker. After blockading endogenous peroxidase with 6% H_2_O_2_ solution (Merck) for 30 min, the slides were incubated in a humidified chamber overnight at 4 °C with the following rabbit primary antibodies: collagen type I (#600-401-103, Rockland, USA), PCNA (clone PC10, cod. M0879, DAKO, USA) and α-SMA (clone 1A4, cod. A2547 Sigma, USA). The slides were then incubated with the complex Super Picture Polymer Detection kit (Life Technologies, USA) for 30 min at 37 °C. The reaction was visualized with 3’3 diaminobenzidine chromogen and counterstained with Harris hematoxylin. The negative controls were performed by omitting the primary antibodies. Counterstaining was performed using Carazzi’s hematoxylin. Slides (*n* = 175) slides were examined under light microcopy, in a Zeiss microscope coupled with a 176 Image Evaluation System (Kontron 300).

### Statistical analysis

Statistical analysis was performed using the analysis of variance (ANOVA).

## Results

### Wound healing and quantitative analysis

In order to evaluate the rLosac effect, even in the early stages of the tissue repair process, lesions were treated immediately after they were induced. The timing established for assessment was 0, 3, 7, 14, and 21 days. During this time period, the majority of events which follow the tissue-repair phases have already taken place [13].

**Fig. 2:**
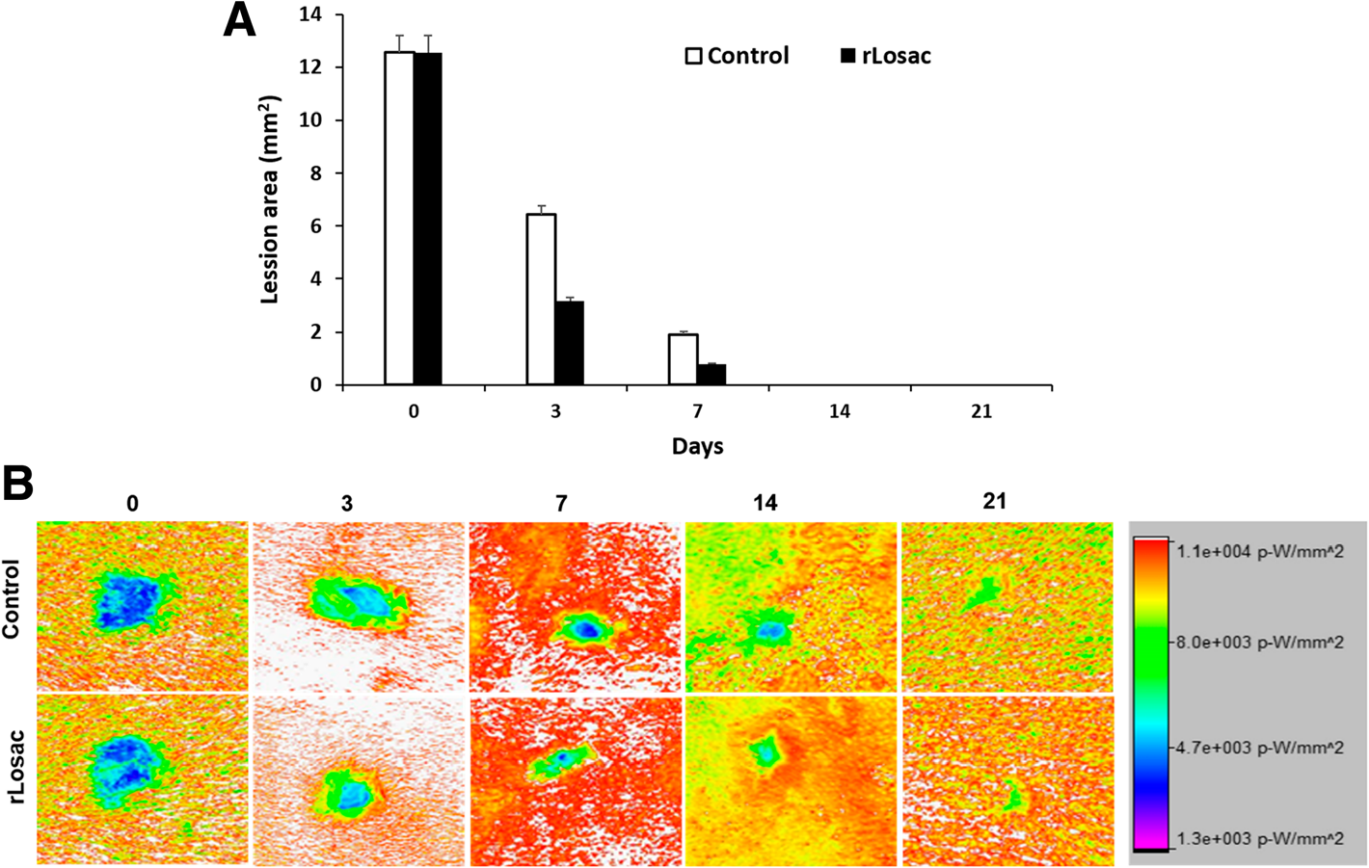
**a** Graph of the optical density of skin lesions on the day of induction, and at 3, 7, 14 and 21 days after surgery. **b** Images were collected for natural fluorescence detection of lesions. The optical density (photons/s/mm^2^) from a fixed region of interest (ROI) was measured

The wounded area measurement is one of the key aspects in the assessment of the healing process given that it provides parameters that can suggest healing improvement or worsening, as well [14]. By day 3, the rLosac administration had decreased the lesion by 31.4%. The same was observed on day 7, when the lesion had diminished by 51.1%. At day 14, a 45.3% decrease was shown in comparison to the control groups. After 21 days of treatment, only a scar could be observed where the lesions were induced (Fig. 2a, b).

### Histological analyses

#### 0 day

**Fig. 3:**
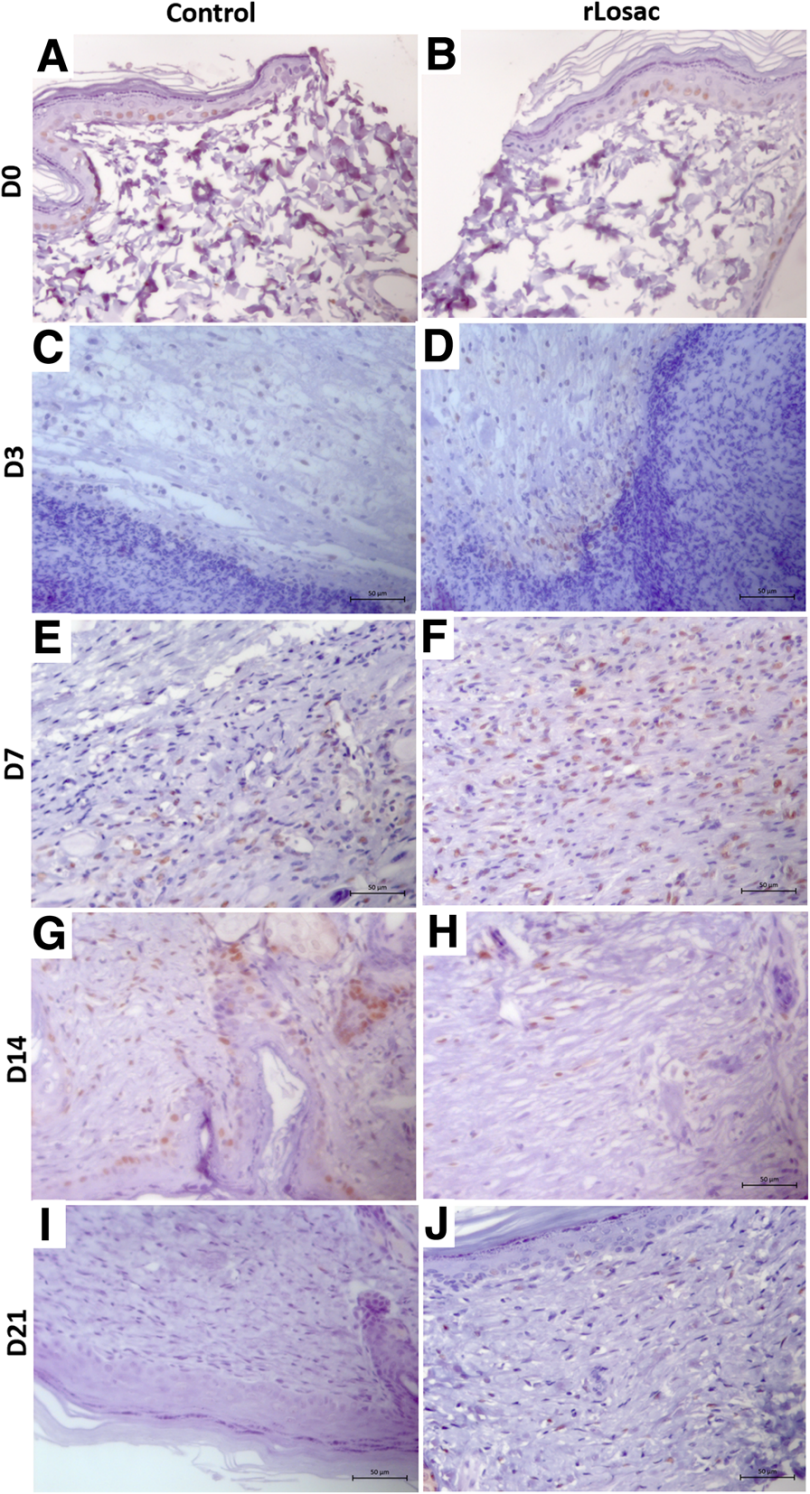
Representative micrographies of PCNA immunohistochemistry. Assessment of scar tissue during the experiment period. Micrographies of scar tissue evaluated at day zero: **a** control group, **b** treated group; day 3: **c** control group, **d** treated group; day 7: **e** control group, **f** treated group; day 14: **g** control group, **h** treated group; day 21: **i** control group, **j** treated group. All areas shown with increases of 40x

In the control group (CG), skin fragment revealed discontinued focus on the epidermis associated with cell debris and fibrin. The other epidermis segments, dermis and adjoining structures remained unspoiled (Fig. 3a). In the treated group (TG), skin fragment displayed discontinued focus on the epithelium, and discrete multifocal acanthosis. The other epidermis segments, dermis and adjoining structures remained unspoiled (Fig. 3b).

#### 3 days

In the CG, ulcerated skin fragments were covered with a pseudomembrane composed of leukocytes and fibrin, discrete multifocal acanthosis, and moderate, acute and chronic mononuclear infiltrate, as well as presence of edema, spreading to deep aspects of the dermis. Inflammatory infiltrate was composed of lymphocytes, plasma cells and foamy macrophages; multinucleated muscle cells were also observed (Fig. 3c). In the TG, ulcerated skin fragments were also covered with a pseudomembrane composed of leukocytes and fibrin. Non-continuous collagen fibers were present in the regions of the ulcerated epithelium and in the panicle of the adjacent epithelium; furthermore, acute and chronic inflammatory infiltrate could be observed. Newly formed blood vessels, fibroblasts reactive to histiocytes, and edema, spreading to the deep aspects of the dermis, were also observed (Fig. 3d).

#### 7 days

In CT: ulcerated skin fragments covered with a pseudomembrane composed of leukocytes and fibrin over the granulation tissue, and discrete regular multifocal acanthosis on the epidermis were observed. The muscular fascia presented moderate mononuclear infiltrate and reactive multinucleate muscle, reduction of lesion size, striation loss, as well as loss of eosinophilia associated with newly formed blood vessels. Absence of continuity of collagen fibers of the deep dermis, and panicle with discrete quantity of unspoiled fibers could be noted amidst an inflammatory focus (Fig. 3e). In TG: moderate regular focal acanthosis associated with orthokeratotic hyperkeratosis on the epidermis could be observed. Discrete area of dermal fibrosis can be detected in association with slight lymphoplasmacytic infiltrate. Newly formed blood vessels, well-organized granulation tissue under the re-epithelialization area, discrete hair follicles with rare neutrophils and discrete macrophages were also visible. The subcutaneous tissue presented a focal area with reactive fibroblasts associated with newly formed blood vessels and discrete lymphoplasmacytic infiltrate, and also a significant presence of fibroblasts and blood vessels perpendicularly distributed (Figs. 3f).

#### 14 days

In GC: regular acanthosis and discrete focal orthokeratotic hyperkeratosis, associated with the area of moderate dermal fibrosis on the epidermis, were observed on the skin fragment; discrete mononuclear inflammatory infiltrate associated with reactive fibroblasts could be seen in the muscular tissue (Fig. 3g). In TG: skin fragments with evidence of initiation of a reepithelialization process were observed, as well as discrete inflammatory and hemorrhagic infiltrate, exudate, and collagen fibers organized in parallel bundles in the dermis. The focal area presented a moderate number of reactive fibroblasts, associated with a discrete quantity of newly formed blood vessels, and lymphoplasmacytic infiltrate. Furthermore, there were skin fragments with signs of regeneration, regular acanthosis, and moderate focal orthokeratotic hyperkeratosis, associated with an area of dermal fibrosis (Fig. 3h).

#### 21 days

In CG: discrete quantity of reactive fibroblasts, associated with a subtle lymphoplasmacytic infiltrate could be observed on the skin fragments. Discrete acanthosis and focal orthokeratotic hyperkeratosis, re-epithelialized skin fragments with orthokeratinized epidermis were observed. Likewise, thin collagen fibers can be seen, some disposed in a parallel fashion and others, in smaller number, perpendicularly disposed (Fig. 3i). In TG: the dermis was observed to be composed of dense connective tissue with moderate quantity of reactive fibroblasts and irregular acanthosis as well as moderate number of collagen fibers having median thickness, which were disposed in both ways, parallel and perpendicular to the epidermis (Fig. 3j).

### Collagen type I, PCNA and α-SMA immunohistochemical evaluation

**Fig. 4:**
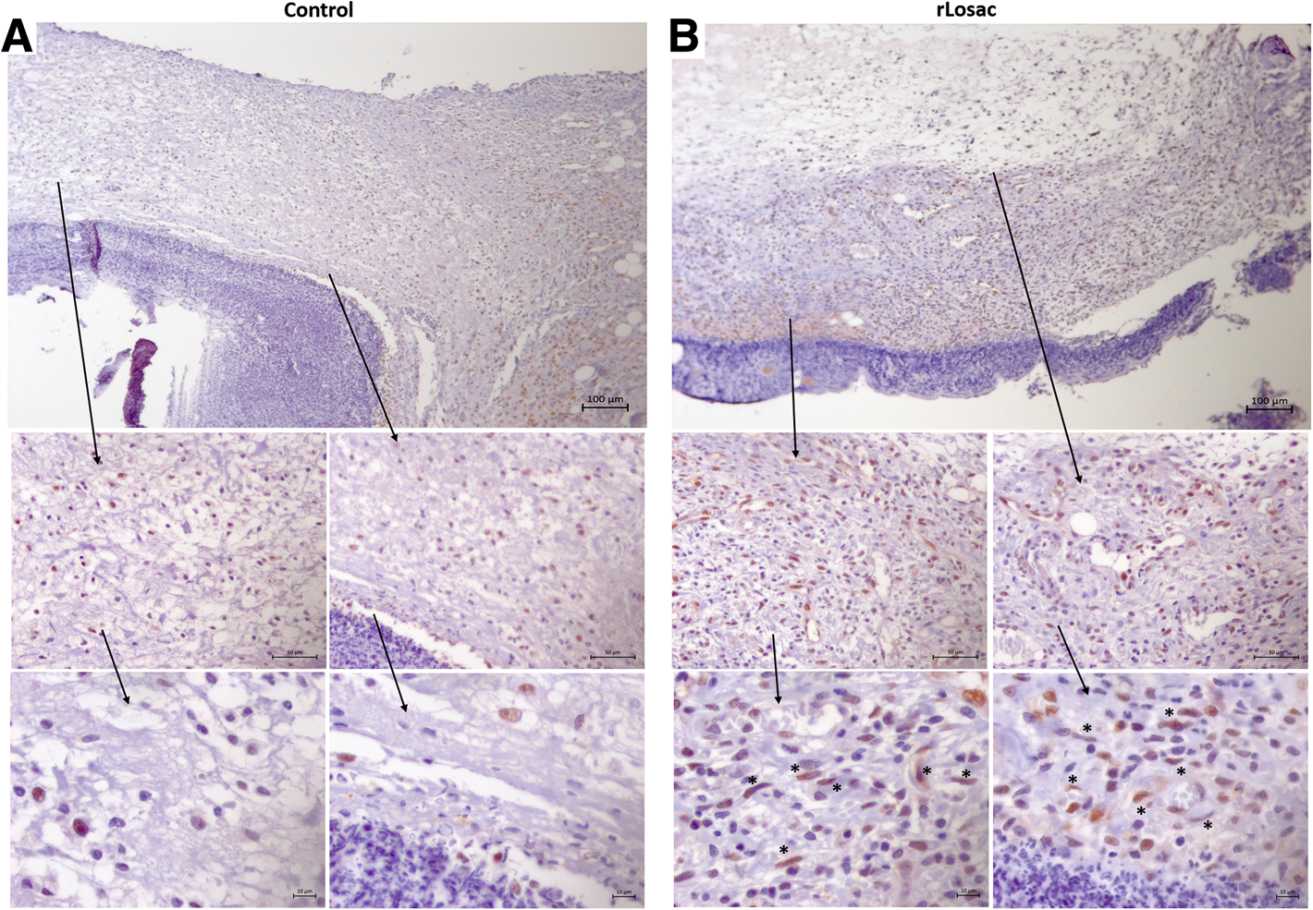
Representative micrographies of immunohistochemistry for PCNA. Assessment of scar tissue by PCNA immunohistochemistry (proliferating nuclear antigen) from the group treated during the experiment period. Micrographies of scar tissue evaluated after day 3 (**a** control group; **b** treated group). All areas shown with increases of 100, 40 and 10x

Proliferating cell nuclear antigen (PCNA) is a cell proliferation marker detected by immunohistochemistry [15]. The PCNA expression pattern was assessed comparatively between the control (Fig. 4a) and treated groups during the healing process. In Fig. 4b, the presence of myofibroblasts and an increase in the PCNA expression can be observed in the epithelial cells and in the activated fibroblasts of the treated group from day 3 onwards, and on the fibroblasts and on epithelial cells on day 7. On day 14, the PCNA expression was evident in the papillary dermis, beneath the lesion area, in the granulation tissue and less expressed in the hypodermis. On day 21, PCNA expression did not differ significantly when compared to the control group.

**Fig. 5:**
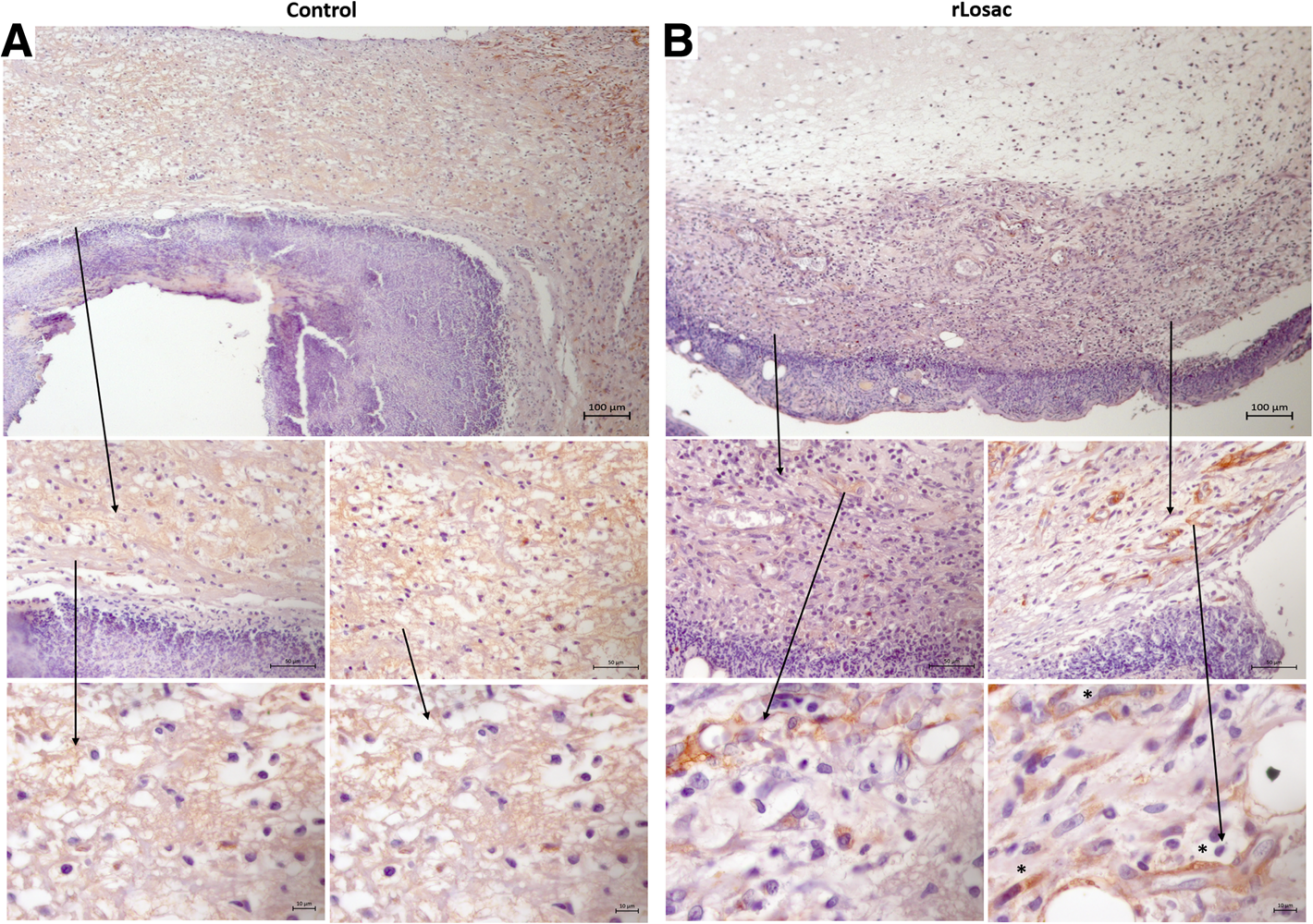
Assessment of scar tissue by immunohistochemistry – collagen type I from the group treated during the experiment period. Micrographies of scar tissue evaluated after day 3 (**a** control group; **b** treated group). All areas are shown at magnifications of 100, 40 and 10x

The deposition of collagen type I differed significantly between the control (Fig. 5a), and the treated groups from day 3 onwards (Fig. 5b), intensifying from day 7, when the treated group presented thicker fibers in different regions of the reticular dermis, culminating in the regular organization of these fibers on day 14, and reepithelialization (only in the treated lesions) after 21 days.

**Fig. 6:**
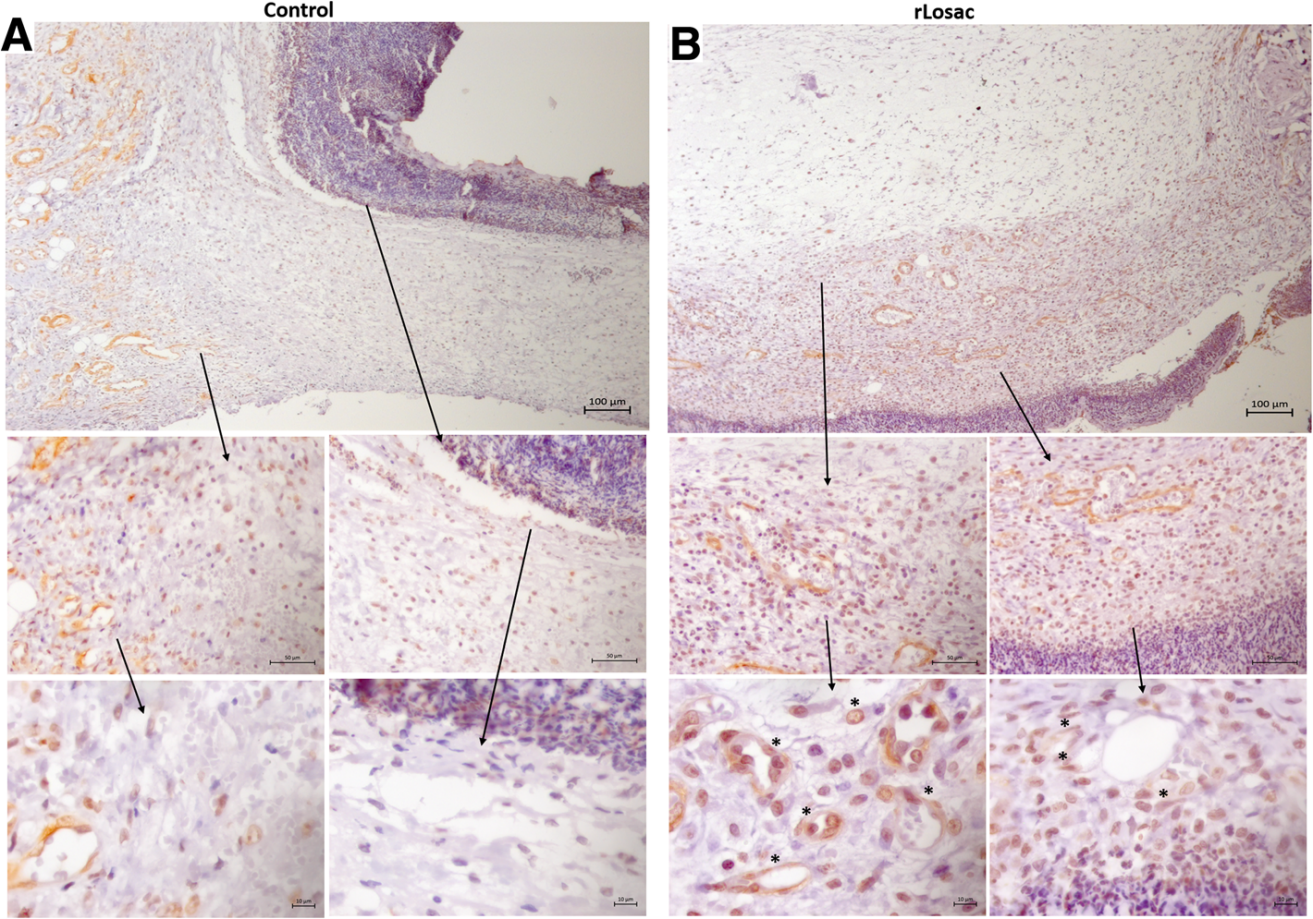
Assessment of scar tissue by immunochemistry – α-SMA from the group treated during experimental period. Micrographies of scar tissue evaluated after day 3 (**a** control group; **b** treated group). All are shown at magnifications of 100, 40 and 10x

α-Smooth muscle actin (α-SMA) is commonly used as a marker of myofibroblast formation and it is regulated by hormones, cell proliferation or wound healing [14]. According to Fig. 6, there was a slight increase in α-SMA expression in the reticular dermis on day 3 of treatment (Fig. 6b). Expression was more intense in the papillary dermis, hypodermis, and in the reticular in the treated groups from day 7 onwards. More intense staining was noted just under the hypertrophied epidermis on day 14. No differences were detected among the groups on day 21.

## Discussion

Wound healing occurs in overlapping steps recognized as hemostasis, inflammation, proliferation and remodeling, which involve interactions between cells and biochemical mediators around the injury site [1]. The present study was carried out to evaluate the possible beneficial effects of rLosac on wound healing following a lesion-induced model in rats. In order to evaluate the effect of rLosac, even though in the early stages of the tissue repairing process, lesions were treated immediately after they were induced. The timing established for assessment was 0, 3, 7, 14 and 21 days, since the majority of events that follow the phases of tissue repairing had already taken place [13].

Myofibroblasts are cells that show morphologic features of both fibroblasts and striated muscle cells. In skin lesions, myofibroblasts migrate from the dermis and other skin tissues surrounding the wounds. These cells are recognized as playing a critical role in generating the contractile force responsible for wound closure and especially for the neo-expression of α-SMA during wound healing [14, 15]. Prolonged presence of fibroblasts with elevated traction force is responsible for wound contraction [16], whereas α-SMA is an actin isoform typical of vascular smooth muscle cells and acts in the increase of collagen deposition in the connective tissue, as well as in tissue remodeling [17, 18]. As to the control group, our results featured an increase in the number of cells resembling fibroblasts and revealed strong immunopositivity for α-smooth muscle actin starting three days after injury, and peaking on days 7 and 14. Then, it gradually decreased until reaching the normal level on day 21. Our findings suggest that an elevated α-SMA expression is sufficient to enhance fibroblast contractile activity in the rLosac-treated lesions.

During tissue repair, fibroblasts and myofibroblasts are recruited to the newly forming tissue and synthesize collagen [16]. Activated fibroblasts present mainly at day 7 in the recovering rLosac-treated wounds may have been responsible for the increase in collagen production observed. In vitro studies using fibroblasts have shown that the protein modulates the expression of extracellular matrix molecules, such as collagen type I, laminin, and fibronectin [12]. Synthesized by fibroblasts, collagen is the most abundant component of the extracellular matrix. During remodeling, collagen becomes increasingly organized. Fibronectin gradually disappears, and hyaluronic acid and glycosaminoglycans are replaced by proteoglycans. Collagen type III is replaced by type I. Type III collagen fibers start to appear by day 2 and 3, followed by the type I collagen fibers that appear by days 6 or 7. The total amount of collagen types I and III increases over time, but the proportion between the types is not constant. The proportion varies from 60% in relation to type III, in the first week after the trauma, to 28% in mature scars [16].

Our immunohistochemical analyses have revealed the presence of collagen fibers in different regions of the reticular dermis from day 3 onwards (Fig. 5), culminating in the organization of these fibers on day 14, and reepithelialization on day 21. Our results have suggested that rLosac improves the replacement of the immature collagen type III by the normal adult type I in treated lesions.

Proliferating cell nuclear antigen (PCNA) is a nuclear protein synthesized in the late G1 and S phases of the cell cycle. Immunohistochemical detection of the protein represents a useful marker for ascertaining the proliferating fraction of cells in tissue specimens [19]. There was significantly enhanced wound closure from days 3 to 7, compared to the control. A histological examination of the tissues on postoperative day 3 revealed that the rLosac treatment increased the number of PCNA immunoreactive cells in the epidermal layer.

Overall, the treatment by rLosac promotes wound healing by increasing the epidermal proliferation and inducing the wound contraction, which are related to the proliferation of myofibroblasts and deposition of collagen.

## Conclusions

The protein rLosac stimulates the activation of fibroblasts, proliferation of epithelial cells, increase of collagen type 1, and decrease of inflammatory infiltrate. The findings presented herein indicate that rLosac is a very promising molecule that is potentially useful as a bioactive agent to develop new formulations for wound healing.

## Abbreviations

cAM: Adenosine 3',5'-cyclic monophosphate
CG: Control Group
cGMP: Guanosine 3',5'-cyclic monophosphate
ECM: Extracellular matrix proteins
EP: Electrostatic potential
Losac: *Lonomia obliqua* Stuart factor activator
PCNA: Proliferating cell nuclear antigen
TG: Treated Group
α-SMA: Smooth muscle actin
